# Using Web-Based Search Data to Study the Public’s Reactions to Societal Events: The Case of the Sandy Hook Shooting

**DOI:** 10.2196/publichealth.6033

**Published:** 2017-03-23

**Authors:** Nir Menachemi, Saurabh Rahurkar, Mandar Rahurkar

**Affiliations:** ^1^ Richard M. Fairbanks School of Public Health Health Policy and Management Indiana University-IUPUI Indianapolis, IN United States; ^2^ Regenstrief Institute Center for Biomedical Informatics Indianapolis, IN United States; ^3^ CareChime Mountain View, CA United States

**Keywords:** Internet, search engine, firearms, health policy, information seeking behavior, public health informatics, gun control debate

## Abstract

**Background:**

Internet search is the most common activity on the World Wide Web and generates a vast amount of user-reported data regarding their information-seeking preferences and behavior. Although this data has been successfully used to examine outbreaks, health care utilization, and outcomes related to quality of care, its value in informing public health policy remains unclear.

**Objective:**

The aim of this study was to evaluate the role of Internet search query data in health policy development. To do so, we studied the public’s reaction to a major societal event in the context of the 2012 Sandy Hook School shooting incident.

**Methods:**

Query data from the Yahoo! search engine regarding firearm-related searches was analyzed to examine changes in user-selected search terms and subsequent websites visited for a period of 14 days before and after the shooting incident.

**Results:**

A total of 5,653,588 firearm-related search queries were analyzed. In the after period, queries increased for search terms related to “guns” (+50.06%), “shooting incident” (+333.71%), “ammunition” (+155.14%), and “gun-related laws” (+535.47%). The highest increase (+1054.37%) in Web traffic was seen by news websites following “shooting incident” queries whereas searches for “guns” (+61.02%) and “ammunition” (+173.15%) resulted in notable increases in visits to retail websites. Firearm-related queries generally returned to baseline levels after approximately 10 days.

**Conclusions:**

Search engine queries present a viable infodemiology metric on public reactions and subsequent behaviors to major societal events and could be used by policymakers to inform policy development.

## Introduction

Nearly 9 out of every 10 Americans have Internet access at home [1] and Web browsing accounts for an average of 23 hours per week that includes activities such as communication, entertainment, news, shopping, and social networking [1,2]. Importantly, searching the Web for information using search engines far surpasses most other types of activities with over 91% of US adults contributing to Web traffic of this nature [3]. Consequently, Web searches generate a vast amount of data in the form of users’ search queries which capture their information-seeking preferences (eg, what they search for) and behavior (eg, what sites they visit). Analysis of this information—a form of infodemiology [4]—could be used to improve our understanding of various issues which in turn can inform policy development.

Infodemiology is an emerging discipline that focuses on analyzing electronic information from the Internet (eg, search queries, social media, and so on) in order to provide information on public health and policy [4]. Previous infodemiology literature has examined Web search query data to evaluate various public health and health care research questions. For example, several studies used Web search query data to identify influenza outbreaks ahead of conventional population detection methods in the United States [5-13] and abroad [14-20], as well as other public health surveillance [21-24]. Researchers have also analyzed search queries for the detection and prevention of adverse drug events, or any other drug related complications [25-27]. Finally, Web-based search logs have been utilized to predict health care utilization and costs following information seeking on search engines [22,28-30]. To our knowledge, no study has examined search data to better understand the public’s sentiments, reactions, and behaviors to major societal events.

We consider major societal events consistent with the “social crises” definition from the crisis management literature. These events are characterized by the severe consequences of the incident, low probability of incident occurrence, and the informational and situational uncertainty that occur among members of the public [31,32]. These situations are inevitably accompanied by collective anxiety, improvised group behaviors, and adaptive collaboration among the public [32-36]. Public mass shooting events share these characteristics; they have a low probability of occurrence, they are followed by lack of reliable information regarding details and consequences of the event, and generate heightened anxiety and public outcry in response to the situation.

The purpose of this paper was to analyze search query data in the context of a major societal event. We decided to study the Sandy Hook Elementary Shooting incident that occurred on December 14, 2012, in Newton Connecticut to determine whether such data can be used to better understand the public’s reactions to such an event. The act of a lone gunman causing the deaths of 20 children and 6 adults received national and international attention, prompting renewed public interest in gun issues [37]. We are interested in understanding how firearm-related information seeking (eg, looking up relevant laws, learning about advocacy) and Web-based behavior (eg, visits to firearm-related retailers) changed immediately after the incident. Understanding these trends will provide insights into how Americans responded to the incident which can enhance societal debates and inform policy development related to firearms.

## Methods

### Data Source and Preparation

We examined deidentified data from Yahoo! search engine queries in a 28-day period before (14-day) and after (14-day) the Sandy Hook shooting incident. Our population consisted of all users of the Yahoo! search engine located in the United States (including Puerto Rico and Mariana Islands) that queried firearm-related searches during the study period. The majority of the information consumed on the Web starts as search queries entered by the user. The choices made by the user in the form of websites they click from the list of populated search results present a much more comprehensive picture of a user’s information needs. Our goal was to use the search query data to evaluate patterns of information seeking regarding firearms and to evaluate broadly the changes in intent based on differences in the content (retail, news, education, and so on) and sources (commercial entities, noncommercial organizations, government entities, and so on) of information sought.

From the complete Yahoo! search query database, we identified all firearm-related queries from November 31, 2012 to December 28, 2012. Queries were text strings consisting of single words or phrases that users typed into the search engine; we identified these using keywords that would match partially or completely with words in the queries. Firearm-related search queries were identified by using keywords in the following categories: Gun type (gun, firearm, handgun, rifle, pistol, revolver, and shotgun), ammunition (ammunition, ammo, and bullets), law related (Brady Act, second amendment of the US constitution), and shooting. In order to choose keywords in each category, we examined Web-based trends of firearm-related search queries for December 2012 using Google Trends. We did this by first examining simpler queries (eg, handgun), and the 10 most correlated searches for these queries. This was repeated recursively with each of the correlated queries until we found no new or correlated searches. This gave us a set of 247 queries that were related to firearms. We wanted our keywords to have the ability to identify these 247 queries as well as any other searches that may be firearm related. Thus our keywords consisted of single words which could identify most firearm-related searches based on complete or partial matches with user queries. As such, our analysis included users’ actual search queries that included keywords in any of the 4 categories.

In addition, we also analyzed the uniform resource locators (URLs) that each individual user clicked from the search results generated by their search queries. First, we identified the domain for each URL that the user clicked; for example, if the user clicked the URL “http://en.wikipedia.org/wiki/Second _Amendment,” the domain was identified as “wikipedia.org.” Next, we categorized these URLs based on the top-level domain (TLD) into commercial entities (.com), noncommercial organizations (.org), government entities (.gov, .state.us, and .mil), educational institutions (.edu), and others (country specific, .pro, .tv, and so on). Including TLDs in our analysis allows us to infer the nature of the organization; for instance, TLDs such as .gov, .mil, and .edu have legal restrictions which prevent them from being used by organizations other than government, military, and educational entities. Moreover, search ranking algorithms are unlikely to place URLs from entities with erroneously used TLDs higher in the search results. These factors allow the use of TLDs to categorize the nature of organizations fairly reliably. Next, each domain was categorized as retail (websites for the purchase of guns, ammunition, and gun accessories; including gun shows), news (websites of newspapers, news channels etc), educational (websites, regardless of TLD, that host information regarding gun safety, gun laws, gun maintenance, and may include websites of gun advocacy groups), showbiz (websites of movies, television shows, music videos, and so on) or “other” which included all remaining uncategorized websites.

The TLD and the content describe different characteristics of the same website and thus examining them together provides a richer understanding of the information seeking patterns. As such we created a variable that assigns a class to each website in the dataset derived from its content category and TLD. Thus, a website with retail content hosted by a commercial entity would be classified as “retail content, .com.” Finally, we created a variable to capture all of the websites owned or affiliated with the National Rifle Association (NRA) as listed on the NRA’s website [38]. Such websites were classified as gun rights advocacy groups. The NRA website also identifies other sites that it categorizes as “antigun lobbying organizations” [39]. We categorized these websites as gun control advocacy groups.

To evaluate the association between the Sandy Hook incident and the nature of information sought, we first examined the distributions of various characteristics of the domains visited by users following the search query (category of keyword, top-level domain, category of the website’s content, and advocacy view of the websites visited). Next, we investigated differences in website characteristics in the period before and after the shooting incident using the website classes. We also examined the percentage change in website visits for each of the characteristics relative to the total websites in the before period to those in the after period. Additionally, we examined the percent change in website visits for each of the characteristics in the after period to the website visit for the same characteristics in the before period.

Finally, it is possible that observed changes in information-seeking behavior over time may be due to the presence of secular or temporal trends and not as a result of the Sandy Hook shooting incident. For example, given that our study period overlapped with the holiday shopping season, one might expect an increase in Web-based shopping activity that can include increases in firearm-related searches, independent of the Sandy Hook incident. To differentiate the shopping activity related increase in search activity from that related to the shooting incident, we included a control query that would be agnostic to the trend due to the Sandy Hook incident but sensitive to the temporal trends of the holiday season. Thus, a query for “bicycle” (and related synonyms) was used as a control search term.

### Limitations

The following limitations must be noted. First, given that Yahoo! search accounted for about 12% of the US search engine market share in December 2012 [40], we recognize that caution must be used before generalizing to the entire US population. Additionally, the Web pages visited by the users may also be associated with result-ranking algorithms which vary by search engines. Since 2011, Yahoo! search is powered by Bing [41] and whereas the exact algorithms are proprietary, evidence suggests that Bing emphasizes keywords (search strings) in ranking search results [42]. Second, our analysis was focused on the query-level (ie, website visited after each search) and not the user level which may include several queries in a given search episode. Third, approximately 30% of all observations consisted of a large number of unique domains occurring with a low frequency and thus could not be classified. Nevertheless, these domains individually accounted for less than 1% of all observations and thus their effect on the findings is likely minimal. Finally, our work represents an exploratory study to examine whether search data can be used for a new purpose. Thus, the existing body of literature provided little guidance on the methods or approaches to analyzing such data. We recognize that future studies may identify additional techniques for analyzing similarly complex data.

## Results

A total of 5,653,588 firearm-related search queries were identified by our keywords in a 28-day period before (14-day) and after (14-day) the Sandy Hook shooting incident. By each search query category (see Table 1), the majority (59.62%; 3,370,523/5,653,588) focused on a gun type (eg, queries with the term pistol, shotgun, or rifle) with the rest focused on the shooting incident (22.47%; 1,270,122/5,653,588), ammunition (16.88%; 954,363/5,653,588), or law related searches (1.04%; 58,580/5,653,588). Based on TLD, users were most likely to visit websites of commercial entities (.com: 88.03%; 4,976,990/5,653,588) followed by noncommercial organizations (.orgs: 6.63%; 374,863/5,653,588) and government entities (.gov, state.us, mil: 1.06%; 59,939/5,653,588). Users most frequently clicked on links that brought them to retail websites (30.33%; 1,714,504/5,653,588), followed by news websites (23.38%; 1,321,706/5,653,588), educational websites (20.32%; 1,148,897/5,653,588), and showbiz websites (2.09%; 118,174/5,653,588). A total of 66,581 websites that users visited could be classified as those of gun rights (68.86%; 45,848/66,581) or gun control (31.14%; 20,733/66,581) advocacy groups. Finally, our control search query for bicycle synonyms yielded 597,859 individual observations during the same study period.

**Table 1:** Characteristics of the search query data. Source: Authors’ analysis of Yahoo! search queries for December 2012.

Variables			Proportion n (%)
Search keywords			
	**Firearm-related (n=5,653,588)**		
		Gun type	3,370,523 (59.62)
		Shooting incidents	1,270,122 (22.47)
		Ammunition	954,363 (16.88)
		Law related	58,580 (1.04)
	**Counterfactual (n=597,859**)		
		Bicycle	597,859 (100.00)
Top-level domain			
	**Firearm-related (n=5,653,588)**		
		Commercial entities	4,976,990 (88.03)
		Noncommercial organizations	374,863 (6.63)
		Government entities	59,939 (1.06)
		Educational institutions	9419 (0.17)
		Other	232,377 (4.11)
Category			
	**Firearm-related (n=5,653,588)**		
		Retail	1,714,504 (30.33)
		News	1,321,706 (23.38)
		Educational	1,148,897 (20.32)
		Showbiz	118,174 (2.09)
		Other or uncategorized	1,350,307 (23.88)
Stance on gun control			
	**Firearm-related (n=66,581)**		
		Gun control advocacy group	20,733 (31.14)
		Gun rights advocacy group	45,848 (68.86)

Bivariate relationships between user search queries and the class of websites visited based on content and TLD are presented in Table 2. In all categories there was an increase in firearm-related search queries in the period after the shooting. Gun type searches which were the most common firearm-related query showed the least relative change after the shooting incident with a 50.06% increase in the proportion of user searches. In contrast, the law category of search queries after the shooting incident had a 535.47% increase in the proportion of searches although it was the least searched. Although users searching for gun types (+61.02%) or ammunition (+173.15%) were more likely to visit retail content on commercial entity websites after the shooting incident, a greater proportion (+1054.37%) visited news content on commercial entity websites for shooting incident searches. Law-related searches, however, had a greater proportion of visits to websites with educational content from noncommercial organizations (+702.70%), commercial entities (+484.20%), and educational institutions (+593.97%). Importantly, when examining changes to bicycle-related search terms (the counterfactual) in the before and after period, we observed a relatively modest decrease in overall searches (−8.64 %).

**Table 2:** Changes in search patterns before and after the Sandy Hook school shooting incident (December 14, 2012). Source: Authors’ analysis of Yahoo! search queries for December 2012.

Search query		n	Before period^a^	After period^a^	Delta %	Cumulative %
**Gun type**							
	Retail content, .com	965,795	370,002 (38.31%)	595,793 (61.69%)	61.02%	28.70%
	News content, .com	595,689	340,883 (57.22%)	254,806 (42.78%)	−25.25%	46.40%
	Educational content, .com	560,295	196,435 (35.06%)	363,860 (64.94%)	85.23%	63.05%
	Educational content, .org	184,979	46,886 (25.35%)	138,093 (74.65%)	194.53%	68.54%
	Other content, .com	774,034	293,009 (37.85%)	481,025 (62.15%)	64.17%	91.54%
	Total	3,365,359	1,345,833 (39.99%)	2,019,526 (60.01%)	50.06%	100%
**Shooting incidents**							
	News content, .com	648,233	51,678 (7.97%)	596,555 (92.03%)	1054.37%	51.45%
	Educational content, .com	94,150	26,941 (28.61%)	67,209 (71.39%)	149.47%	58.93%
	Educational content, .org	54,830	7911 (14.43%)	46,919 (85.57%)	493.09%	62.28%
	Showbiz related content, .com	44,135	15,732 (35.65%)	28, 403 (64.35%)	80.54%	66.78%
	Other content, .com	319,892	98,564 (30.81%)	221,328 (69.19%)	124.55%	92.18%
	Total	1,259,817	236,050 (18.74%)	1,023,767 (81.26%)	333.71%	100%
**Ammunition**							
	Retail related content, .com	609,246	163,272 (26.80%)	445,974 (73.20%)	173.15%	63.85%
	Educational content, .com	95,687	31,256 (32.66%)	64,431 (67.34%)	106.14%	73.88%
	News content, .com	61,025	10,849 (17.78%)	50,176 (82.22%)	362.49%	80.28%
	Other content, .com	100,711	36,104 (35.85%)	64,607 (64.15%)	78.95%	90.83%
	Total	954,158	268,670 (28.16%)	685,488 (71.84%)	155.14%	100%
**Laws**							
	Educational content, .org	25,429	2817 (11.08%)	22,612 (88.92%)	702.70%	43.41%
	Educational content, .com	12,473	1823 (14.62%)	10,650 (85.38%)	484.20%	64.70%
	Educational content, .edu	3422	431 (12.59%)	2991 (87.41%)	593.97%	70.54%
	News content, .com	2663	383 (14.38%)	2280 (85.62%)	495.30%	75.09%
	Other content, .com	8978	1502 (16.73%)	7476 (83.27%)	397.74%	90.41%
	Total	58,580	7965 (13.60%)	50,615 (86.40%)	535.47%	100%
**Bicycle**							
	Retail related content, .com	142,209	75,551 (53.13%)	66,658 (46.87%)	−11.77%	24.08%
	Educational content, .com	87,964	45,550 (51.78%)	42,414 (48.22%)	−6.88%	38.98%
	Other content, .com	266,220	137,068 (51.49%)	129,152 (48.51%)	−5.78%	84.06%
	Other content, .other	23,605	12,254 (51.91%)	11,351 (48.09%)	−7.37%	88.06%
	Other content, .org	19,957	10,233 (51.28%)	9724 (48.72%)	−4.97%	91.44%
	Total	590,530	308,603 (52.26%)	281,927 (47.74%)	−8.64%	100%

^a^Indicates ± 14 days from the Sandy Hook event.

Table 3 presents the bivariate relationship between the time period and the advocacy view (gun rights vs gun control) of the websites visited by users following search queries for each category of firearm-related searches. Search results increased in all categories in the period after the shooting incident (range: 79.94%-418.39%). The majority of search queries for gun type (67.92%; 40,069/58,998), shooting incident (84.55%; 2490/2945), and ammunition (93.16%; 2316/2486) resulted in users visiting websites of gun rights advocacy groups, whereas those searching for laws were more likely to visit websites of gun control advocacy groups (54.79%; 1179/2152). On the whole, Web-based users had an increase of between 285.71% and 660.58% of visiting gun control advocacy group websites after the shooting incident.

**Table 3:** Bivariate relationship between time period and the advocacy view of the websites visited by users for each category of firearm-related searches. Source: Authors’ analysis of Yahoo! Search queries for December 2012.

Search query		n (%)	Before^a^	After^a^	Delta %
**Gun type**						
	Gun rights	40,069 (67.92)	28.29%	71.71%	153.53%
	Gun control	18,929 (32.08)	14.97%	85.03%	467.93%
	Total	58,998	24.01%	75.99%	216.42%
**Shooting incidents**						
	Gun rights	2490 (84.55)	39.04%	60.96%	56.17%
	Gun control	455 (15.45)	17.58%	82.42%	368.75%
	Total	2945	35.72%	64.28%	79.94%
**Ammunition**						
	Gun rights	2316 (93.16)	36.44%	63.56%	74.41%
	Gun control	170 (6.84)	20.59%	79.41%	285.71%
	Total	2486	35.36%	64.64%	82.82%
**Laws**						
	Gun rights	973 (45.21)	21.69%	78.31%	261.14%
	Gun control	1179 (54.79)	11.62%	88.38%	660.58%
	Total	2152	16.17%	83.83%	418.39%

^a^Indicates ±14 days from the Sandy Hook event.

**Figure 1:**
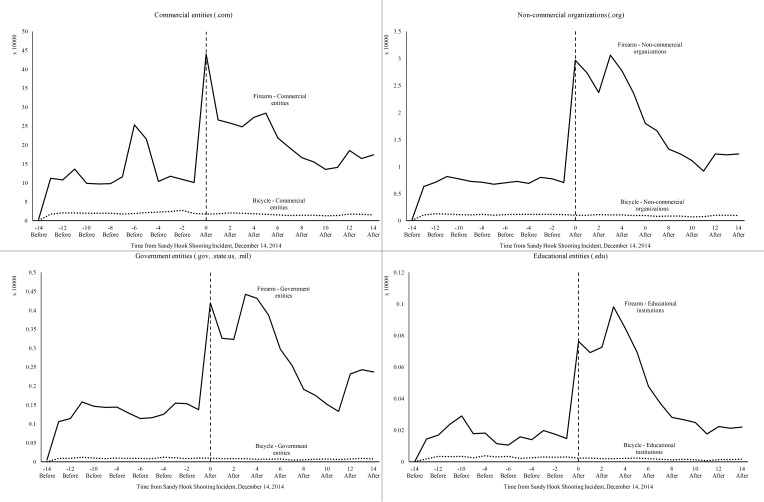
Websites visited following firearm-related and bicycle-related search queries by top-level domain. Source: Authors’ analysis of Yahoo! Search queries for December 2012.

**Figure 2:**
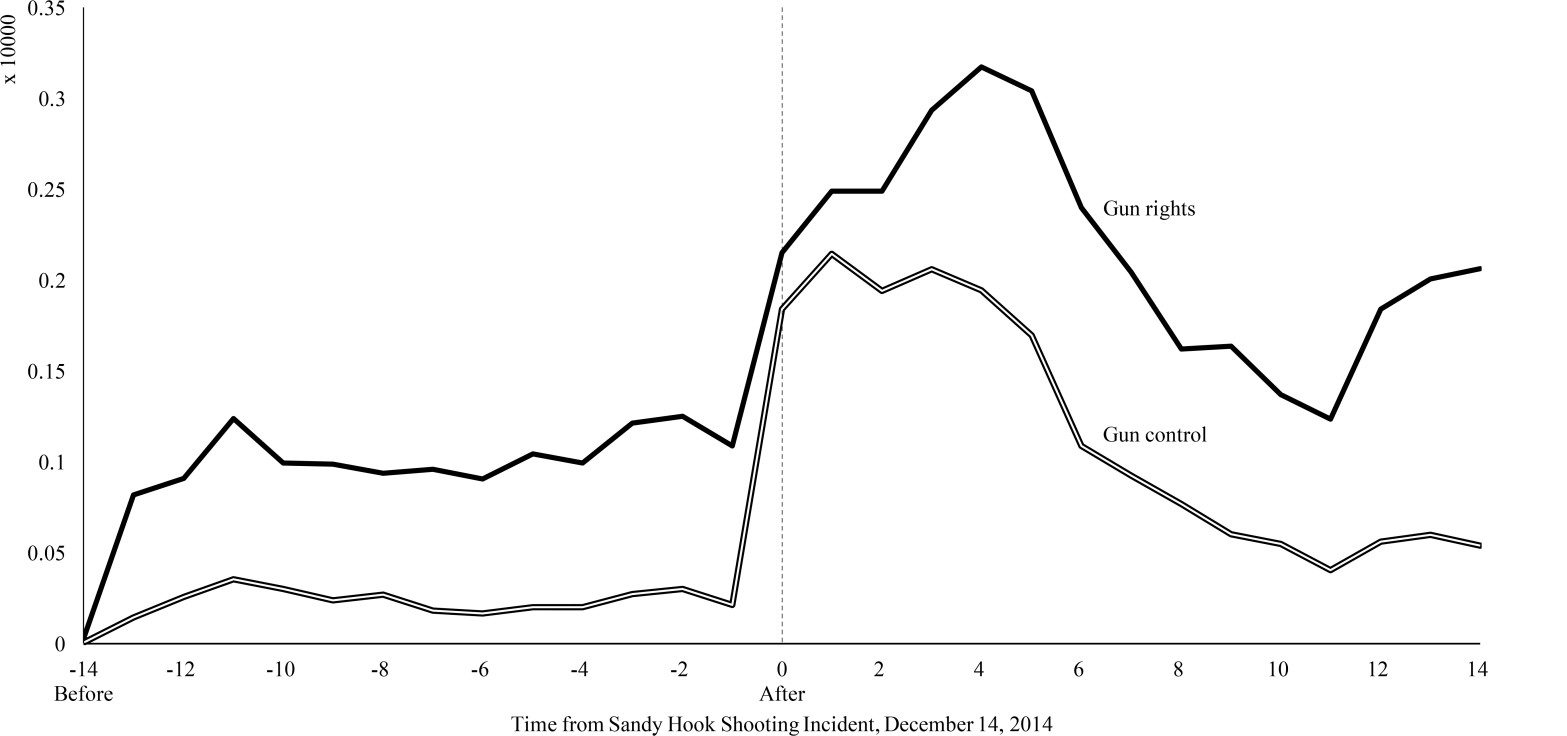
Trends in website visits by advocacy view. Source: Authors’ analysis of Yahoo! search queries for December 2012.

Figure 1 presents the trend data graphed in the before and after period for firearm-related and bicycle-related searches for 4 categories of TLDs. As can be seen, Web traffic as a result of firearm-related search queries saw a sharp increase corresponding to the Sandy Hook shooting incident for domains of commercial entities, educational institutions, government entities, and noncommercial organizations. Additionally, depending on the TLD, a relatively smaller peak in Web traffic is seen at days 6 and 11 before the shooting incident following firearm-related searches, with the greatest increase seen for .com domains. Conversely, bicycle-related searches during the same period appear relatively unchanged. Figure 2 presents the trend data graphed in the before and after period for firearm-related search queries for advocacy view. Websites of both gun control and gun rights advocacy groups saw a sharp increase in traffic corresponding to the shooting incident following firearm-related searches. The traffic decreased slowly for both over the after period with slight increase in traffic at day 11.

## Discussion

### Principal Findings

One of the key findings of our analysis was that firearm-related searches more than doubled immediately after the Sandy Hook shooting incident in contrast to the control searches for “bicycle” which showed a small change with a decrease in the number of searches in the after period. This finding suggests that Web-based user search queries capture the immediate change in public interest following events of the nature of Sandy Hook shooting and can thus potentially serve as real-time indicators of the public psyche.

Overall, retail websites were the most visited websites following searches for gun types and ammunition. A salient finding was that gun type and ammunition searches had a 2-fold to 3-fold increase after the shooting incident. Furthermore, although it may seem natural to expect a greater interest in news articles following a major societal event, retail website visits had the highest and second highest increase after the shooting incident for gun type and ammunition searches, respectively. This finding may possibly be the result of a heightened interest in purchasing firearms and/or ammunition for one’s protection against the apparent public safety concerns raised by the mass shooting [43]. Additionally, it is possible that some individuals may anticipate an increase in regulatory control over access to firearms as a ramification of the Sandy Hook incident and as such prompt purchase of firearms before any such legislative action is passed.

Furthermore, there was a 6-fold increase in law-related firearm queries in the period immediately after the shooting. Importantly, these were the least likely searched terms in the before period and noted the greatest percent increase in the after period. This increased interest may be due—in part —to the purchase-related search or inquiry conducted by the potential firearm and ammunition buyers discussed above, not to mention the renewed interest in the gun-policy debate after Sandy Hook. Interestingly, most users seeking law-related information were interested in educational information and chose websites of noncommercial organizations, commercial entities, or educational institutions. From the advocacy perspective, more people visited websites of gun rights groups than did the gun control groups. However, despite gun control websites forming a lower proportion of all websites supporting an advocacy stance, they experienced the greatest percent increase from before to the after period. This trend was seen in all categories of firearm-related searches, with Web traffic to gun control advocacy groups exhibiting between almost 4-fold and 6-fold increase in the after period.

In addition to the trends discussed previously, a key feature of user searches and the subsequent URL clicks was that in all categories users were far less likely to choose content from a government entity. For example, even though the majority of the law-related searches are directed toward educational content, users are more likely to choose noncommercial organizations (including gun control or gun rights advocacy groups), commercial entities, and educational institutions as their preferred sources of information. The nature of advocacy groups is such that they exist to influence stakeholder decision to align with their agenda and therefore, the resulting conflict of interest may be an impediment to providing unbiased information. Thus, it is also likely that users seeking information about gun laws may obtain this information from websites of advocacy groups.

Our analysis of user search query data presents several key implications from a policy perspective. First, as stated above, user search queries present a valuable real-time indicator of the attitudes of the population as shown by the effect of the Sandy Hook shooting incident. In fact, the spike seen 6 days before the Sandy Hook event corresponds to 2 news stories: one on December 7, when supermarket employees found a handgun in frozen meat [44] and another on December 9, when a 7-year-old boy was fatally shot in the parking lot of a gun store [45]. Similarly, the spike seen around day 12 corresponds with the much publicized advocacy speech given by a prominent American sportscaster on television [46]. These spikes highlight user search queries as a timely measure of the public’s reaction to societal events. The time period immediately after a major event is characterized by heightened awareness and information-seeking behavior that may not be representative of public action during normal states (eg, buying firearms at twice the regular prices [43]). Indeed, Oh et al note that “rumormongering” is common after major societal events including shooting events [32]. On the one hand, this may indicate that policymakers should consider the timing of their actions noting that while a societal event can trigger interest in a topic, it ironically may not the best time to debate major tenets of policy change. On the other hand, some observed behavior may be due to fears arising from misinformation. For example, the increased purchase-related queries in our findings corroborate increased firearm sales due to fear of increased gun control legislation [43].

Second, it is possible that people are accessing information sources with either commercial or advocacy-related interest, at the same time being far less likely to choose content from government and educational institution websites. This may be because websites of government and educational institutions rank lower in the search results compared with those of commercial and advocacy interest groups. Although search engine optimization (SEO) may play a role in the higher ranking of commercial and advocacy interest websites, it is also possible the information presented by government and educational institutions may be less accessible. This may be due to suboptimal website design, jargon-filled language, poor SEO, lack of up-to-date information, and so on. Policy efforts should focus on providing reliable information as well as improved dissemination of this information by government institutions. Government entities may collaborate with educational institutions toward the creation of information portals focused on dissemination of accurate, timely, and high-quality information that is easy to understand. Furthermore, resources allocated toward making the public aware of these portals as well as on SEO may ensure that these websites rank higher in search results and thus visited more often.

Finally, the increased interest generated by the shooting incident appears to start tending toward normal levels around day 10, eventually returning to the levels before the shooting. This indicates that the increased interest generated due to incidents such as Sandy Hook presents a short window in which to form the public’s opinion. As discussed previously, this may not present the best opportunity to engage in public debate due to the increased anxiety and fear following these events. Whether this fear was driven by the need to protect oneself or the possibility of losing the right to purchase a firearm, it is unlikely that political sentiment for policy change will be easy to accomplish when fear is driving some stakeholder’s perspectives. Instead, policymakers should consider preemptively addressing some of the anticipated fears by implementing targeted campaigns that focus on specific groups of individuals. A recent US study reported that 3 percent of the US population owns nearly half of all firearms in the country with an average of 17 firearms each [43,47-49]. The median firearm ownership, however, remains at 1 to 2 firearms per owner. These individuals are likely to indulge in firearm purchases [43] after events such as the Sandy Hook shooting. Furthermore, given that personal protection against other people remains the most prevalent reason for firearm ownership in the US [47], mass shooting events may also motivate those on the fence to purchase firearms. As such, targeted campaigns that focus on these groups of individuals in order to allay fears and reduce reactionary purchase of firearms may help achieve some policymaker’s goals of lower rates of firearm ownership.

### Conclusions

Our findings enabled us to identify directions for future research; web browsing choices and attitudes toward firearms may be affected by numerous other factors. As such, it may be valuable to examine the differences between attitudes toward firearms based on state characteristics such as political affiliation, socioeconomic status, and gun ownership. It may also be interesting to look at ordered queries nested within each deidentified user based on the order in which the user clicked each URL to provide richer data on users’ search intent. Search query data presents a valuable infodemiology metric of near real-time analysis of peoples’ attitudes and responses to major societal events. We believe future studies can employ the use of other search query datasets possibly with active user participation to examine the impact of society events over a longer period of time.

## Abbreviations

NRA: National Rifle Association
SEO: search engine optimization
TLD: top level domain
URL: uniform resource locator
